# An Updated Meta‐Analysis of Randomized Controlled Trials Comparing the Analgesic Efficacy of Intercostal Nerve Block Versus Erector Spinae Plane Block in Thoracic Surgery

**DOI:** 10.1155/anrp/7833175

**Published:** 2026-11-03

**Authors:** Muhammad Shaheer Bin Faheem, Syeda Umbreen Munir, Kanz Ul Eman Maryam, Sabahat Ul Ain Munir Abbasi, Sumaya Samadi

**Affiliations:** ^1^ Department of Medicine and Surgery Karachi Institute of Medical Sciences Karachi Sindh Pakistan; ^2^ Department of Medicine Karachi Medical and Dental College Karachi Sindh Pakistan duhs.edu.pk; ^3^ Department of Medicine Foundation University Medical College Islamabad Pakistan fui.edu.pk; ^4^ Department of Medicine Allama Iqbal Medical College Lahore Pakistan aimc.edu.pk; ^5^ Department of Medicine Kabul University of Medical Sciences “Abu Ali Ibn Sina” Kabul Afghanistan duhs.edu.pk

**Keywords:** : analgesic efficacy, erector spinae plane block, ESPB, ICNB, intercostal nerve block, meta-analysis, postoperative pain management, thoracoscopic surgery

## Abstract

**Background:**

ESPB, an ultrasound‐guided fascial plane block, gained considerable attention as a regional anaesthetic because of its longer duration of action, favourable safety profile, and multidermatomal analgesic effect. ICNB, however, is associated with a relatively short duration of action, often requiring repeated dosing and adjunctive systemic analgesics.

**Methods:**

The methodology of this meta‐analysis adhered to the PRISMA 2020 guidelines and included randomized controlled trials comparing ESPB with ICNB in thoracoscopic surgery. A systematic PubMed, Cochrane, and ClinicalTrials.gov search was performed that reviewed the publications available till March 16, 2025. Studies were screened, data were extracted, and two reviewers independently evaluated the risk of bias using the Cochrane ROB 2 tool. Dynamic pain at 48 h was the primary outcome; the secondary outcome was analgesic use. Standardized mean differences (SMDs) or mean differences (MDs), as appropriate, with 95% confidence intervals were computed. Version 5.4 of Review Manager (RevMan) was used to process the statistical analysis, with a random‐effects model where necessary.

**Results:**

We identified 444 records, out of which 6 randomized controlled trials were part of this meta‐analysis, totalling 353 cases: 176 being part of the ESPB group, while the remaining 177 constituted the ICNB group. For the principal outcome of dynamic pain score at 48 h postoperatively, combined analysis from five studies (*n* = 295) revealed a statistically significant lowered pain score with ESPB in contrast to ICNB (SMD = −0.34, 95% CI: −0.57 to −0.10, *p* = 0.004; *I* ^2^ = 0%). For postoperative analgesic use, all six studies (*n* = 353) data showed insignificant group difference (MD = 0.14, 95% CI: −1.04 to 1.32, *p* = 0.81; *I* ^2^ = 57%).

**Conclusion:**

This study suggests that ESPB contributed to a reduction in pain scores at 48 h after thoracoscopic surgery compared with ICNB, yet both methods exhibited equivalent postoperative analgesic consumption. These findings show that ESPB can be a preferable option for prolonged postoperative analgesia; however, additional well‐designed studies are needed to corroborate the evidence base.

## 1. Introduction

In the past, open thoracotomy was widely used to access the thoracic cavity for various respiratory procedures. But now it has largely been replaced by thoracoscopic surgery because of its association with fewer perioperative risks and postoperative complications. Despite its advantages over open thoracotomy, chronic postoperative pain following thoracic surgery remains a persistent challenge, affecting 25%–60% of patients, making effective perioperative pain management a serious clinical concern [1].

The landscape of perioperative pain management has undergone multiple shifts in recent years. The emphasis, especially, has been on developing multimodal, opioid‐sparing strategies that favour reduced complications and quick patient recovery [2]. As minimally invasive approaches like video‐assisted thoracoscopic surgery (VATS) have become prevalent, anaesthetic techniques are also evolving in parallel to support enhanced recovery pathways, and patient‐centred care.

In this context, the development of regional anaesthesia techniques has represented a major advancement by providing site‐specific analgesia while minimizing systemic adverse effects. The intercostal nerve block (ICNB) provides direct nerve infiltration; however, its short duration of action, operator dependence, and need for multiple‐level injections limit its widespread use [3, 4]. In contrast, the more recently described erector spinae plane block (ESPB), performed under ultrasound guidance, exploits fascial plane spread to provide multidermatomal analgesia from a single injection site [5, 6].

Several studies have compared the analgesic efficacy of ESPB and ICNB in patients undergoing thoracoscopic surgery [7–12]. As anaesthesiologists and surgeons seek the least invasive and most effective options for pain control during thoracoscopic procedures, it is important to assess how these two approaches were compared based on postoperative pain scores, analgesic requirements, and complications. Although previous studies have evaluated the analgesic efficacy of ESPB and ICNB, important differences still exist between prior publications and the present study. The previous meta‐analysis by Ma et al. directly compared the ESPB and ICNB in thoracoscopic surgeries using the evidence available at that time, whereas the present study incorporates additional randomized controlled trials (RCTs) published subsequently and provides an updated synthesis of available evidence [13]. In addition, a recently published article by Wu et al. in BMJ Open describes a protocol for a systematic review and meta‐analysis of ESPB vs. ICNB rather than reporting completed pooled results [14]. Furthermore, a previous network meta‐analysis evaluated multiple regional analgesic methods following thoracic surgery, including ESPB vs. ICNB, but was not specifically focused on direct comparison between both of them [15]. Therefore, this study provides an updated direct comparison of ESPB and ICNB in thoracoscopic surgeries, with a special focus on postoperative pain at 48 h, postoperative analgesic consumption, clinical relevance, and potential sources of heterogeneity.

## 2. Methods

### 2.1. Registration and Protocol

The concerned review and meta‐analysis was documented in the PROSPERO database under registration number 10,44,331. A separate review protocol was not published.

### 2.2. Database Search and Study Selection

Observing strict adherence to the 2020 guidelines provided by the Preferred Reporting Items for Systematic Reviews and Meta‐Analyses (PRISMA 2020), this meta‐analysis has been conducted [16]. The reviewers thoroughly searched the databases of PubMed, Cochrane, and Clinicaltrials.gov from their inception to March 16, 20,265. The search terms included: (erector spinae plane block OR ESPB) AND (intercostal nerve block OR ICNB) AND (thoracoscopic OR thoracoscopy). The comprehensive PubMed search protocol is listed in Supporting Table S1. The bibliographic references of all included publications were also searched manually for any further germane articles.

Additionally, the following inclusion criteria were applied: (1) studies involving participants undergoing thoracoscopic surgery; (2) the intervention group received ESPB; (3) the comparison group received ICNB; and (4) the study design was an RCT. Both reviewers independently conducted title and abstract screening. Disagreements were resolved through discussion and consensus. Inter‐reviewer agreement statistics, such as Cohen’s kappa, were not formally calculated. The finalized PRISMA 2020 checklist is also provided as Supporting Figure S1.

### 2.3. Data Extraction

Two reviewers individually evaluated the available literature for eligible publications. After replicates were withdrawn in Rayyan.ai, titles and abstracts were then independently screened, and full‐text articles were evaluated against the predetermined inclusion criteria. The two reviewers, after extracting the relevant data using a predesigned data extraction form, also cross‐verified it for precision and consistency. Extracted information included study descriptors (first author’s name, year of publication, and study location); baseline demographics (sample size, mean age with standard deviation [SD], gender distribution, body mass index [BMI], and American Society of Anaesthesiologists [ASA] physical status classification); and outcomes (dynamic pain intensity at 48 h postoperatively, analgesic consumption).

The primary outcome was dynamic postsurgical pain at 48 h, measured using the Visual Analogue Scale (VAS) or Numeric Rating Scale (NRS) as reported in each study, while the secondary outcome was total postoperative analgesic consumption, reported as per study‐specific units.

Only results corresponding to the 48‐h time point were extracted, as it represents a clinically relevant phase of postoperative recovery during which patients continue to experience significant movement‐related pain following thoracoscopic surgery. Prior literature has demonstrated that the pain intensity remains substantial within the first 48 h postoperatively, especially during mobilization and respiratory effort [17, 18]. This time point is also the most frequently reported across the trials included in this meta‐analysis, allowing for standardized comparisons of outcomes. Moreover, disagreements were settled by discussion and consensus. We also communicated with corresponding authors of two studies to obtain missing data; one author responded and provided additional information, while we received no response from the other, corresponding to a 50% response rate. When no response was received, we used statistical methods to calculate the mean and SD from the reported median and interquartile range, adjusting for skewness when necessary [19]. Funding sources were recorded where reported.

### 2.4. Quality Assessment

The Cochrane Risk of Bias 2 (ROB 2) tool, designed especially for RCTs, was used to assess the quality and risk of bias of the included studies [20]. The ROB 2 tool assesses five major categories of bias: (1) bias due to the randomization process, (2) bias due to deviations from the planned interventions, (3) bias due to missing outcome data, (4) bias in the measurement of the outcome, and (5) bias in the selection of the reported result.

Two reviewers independently reviewed each study, and any discrepancies between reviewers were settled through mutual discussion and with the help of intervention by and consultation with the third reviewer. Risk of bias judgements were grouped as ‘Low risk’, ‘Some concerns’, or ‘High risk’ based on the ROB 2 tool’s signalling question responses. The evaluation was performed using the ROB 2 Excel tool, and traffic‐light plots and weighted summary graphs were produced for visualization, providing a snapshot of risk across the studies. Several studies that showed a high risk of bias in more than one area underwent sensitivity analysis to assess the effect of bias on meta‐analytic estimates. In this way, the results were reliable and robust, especially for the primary outcome of dynamic pain at 48 h, which is prone to measurement and reporting variations.

### 2.5. Statistical Analysis

For continuous data, we estimated the standardized mean difference (SMD) for pain outcomes and mean differences (MDs) for analgesic consumption, with 95% confidence intervals (CIs). We used the Chi‐squared (*χ* ^2^) test to assess statistical heterogeneity among the studies included in the review and quantified it using the *I* ^2^ statistic. A value of *I* ^2^ higher than 50% was taken as evidence of substantial heterogeneity. For significant heterogeneity, a random‐effects model was used. A leave‐one‐out approach was used to conduct the sensitivity analyses for investigation of possible sources of heterogeneity and to assess the influence of individual studies on the overall pooled effect.

As the number of included studies was small (*n* = 6), subgroup analysis was not conducted, as these analyses require a greater number of studies to yield significant conclusions. Furthermore, publication bias was not evaluated, as less than 10 studies were featured, which renders funnel plot asymmetry and corresponding statistical tests unreliable. Version 5.4 of Review Manager (RevMan) was used to process all the statistical analysis.

## 3. Results

We conducted a systematic review and meta‐analysis to compare the analgesic efficacy between erector spinae and ICNBs in patients undergoing thoracoscopic surgery. A total of 444 records were initially retrieved through electronic database searches of PubMed, Cochrane, and Clinicaltrials.gov. After removal of 7 duplicates using Rayyan.ai, 437 articles were screened based on their titles and abstracts. Of these, 431 articles were withdrawn, and the remaining 6 full‐text articles were evaluated for validity through full‐text screening. After a thorough review, 4 studies were excluded, and 2 new publications finally became part of this systematic review and meta‐analysis. As this is an updated Meta‐analysis, we included 6 studies: 4 from the previous Meta‐analysis and 2 newly identified studies. A PRISMA flow diagram of the study inclusion method is demonstrated in Figure 1.

**FIGURE 1:**
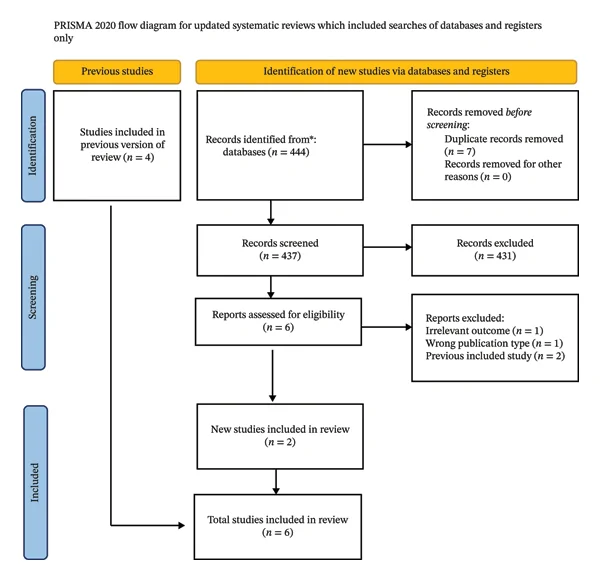
Flow diagram for the study inclusion method used in the meta‐analysis.

The six articles, authored from 2020 to 2024, encompass a broad demographic and clinical spectrum, with variations in surgical procedures, regional block types, and analgesic regimens. Most studies have an adult patient population with a balanced distribution of male and female participants.353 cases were selected for the meta‐analysis, with 176 receiving an ESPB and 177 receiving an ICNB. There were no significant reported losses to follow‐up across any of the studies. Comprehensive details of each study are tabulated in Table 1.

**TABLE 1:** Baseline characteristics of the studies included.

ESPB group								ICNB group					
Study ID	Year	*N*	Age (year)	Male, *n*	BMI (kg/m^2^)	ASA physical status (I/II)	Method	*N*	Age (year)	Male, *n*	BMI (kg/m^2^)	ASA physical status (I/II)	Method
Sung	2024	50	58 ± 14	26	24.1 ± 2.6	4/38	ESPB with 20 mL of 0.375% ropivacaine	50	60 ± 11	21	23.5 ± 2.7	4/39	ICNB with 20 mL of 0.5% levobupivacaine across five intercostal spaces
Gams	2023	25	69.44 ± 8.20	17	25.72 ± 4.18	0/14	ESPB with 20 mL 0.5% levobupivacaine bolus, followed by 0.2% ropivacaine every 4 h	25	70.2 ± 8.76	13	27.03 ± 3.64	1/11	ICNB with 20 mL 0.5% levobupivacaine across 6 intercostal spaces
Turhan	2021	35	53.31 ± 9.03	19	24.38 ± 1.57	13/19	ESPB with 20 mL 0.5% bupivacaine	36	52.47 ± 10.82	19	24.13 ± 1.98	12/19	ICNB with 20 mL 0.5% bupivacaine (4 mL each at T4–T7)
Hort	2021	12	71.33 ± 9.00	5	28.34 ± 4.03	NR	ESPB with 30 mL of 0.125% bupivacaine	12	66.50 ± 8.44	2	25.10 ± 5.1	NR	ICNBs at T4–T11 using 5 mL of 0.25% bupivacaine with epinephrine per level at the end of surgery (max 2.5 mg/kg)
Fiorelli	2020	30	67.47 ± 9.34	18	25.32 ± 4.24	0/25	ESPB with 20 mL of 0.75% ropivacaine	30	68.37 ± 11.88	14	25.57 ± 4.01	0/14	ICNB with 20 mL of 0.75% ropivacaine injected into intercostal spaces from T4 to T8
Chen	2020	24	53.3 ± 11.6	13	22.9 ± 3.1	10/14	ESPB with 20 mL of 0.375% ropivacaine	24	58.1 ± 7.0	15	23.5 ± 2.4	9/15	ICNB with 4 mL of 0.375% ropivacaine injected into intercostal spaces from T4 to T9

*Note:* ASA values are presented as the number of patients classified as ASA I/ASA II, as reported in the original studies. NR, not reported in the original study.

The Cochrane Risk of Bias (RoB 2.0) tool for RCTs was used to evaluate the quality of these studies. The risk of bias assessment indicated that of the six included studies, 1 had a low risk of bias, 3 had a moderate risk, while 2 exhibited a high risk of bias. A comprehensive breakdown of quality assessments is provided in the Supporting Table S2.

### 3.1. Pain Scores at 48 Hours (Dynamic):

Five out of six studies reported postoperative pain scores comparing the ESPB and ICNB. The data were available for a total of 295 patients (148 in ESPB, 147 in ICNB). The pooled analysis revealed a statistically significant mitigation in pain scores favouring ESPB (SMD = −0.34, 95% CI: −0.57 to −0.10, *p* = 0.004), indicating that patients who received ESPB experienced lower postoperative pain than those who were injected with ICNB. No significant heterogeneity (*I* ^2^ = 0%, *p* = 0.58) was noticed throughout the selected studies, as shown in Figure 2.

**FIGURE 2:**
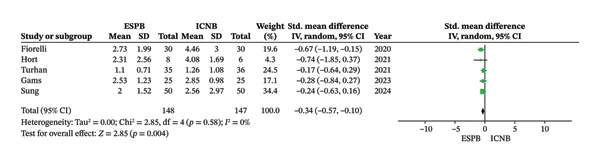
Forest plot of pain scores at 48 h (dynamic).

### 3.2. Analgesic Consumption

All six studies comprising 353 patients (176 in the ESPB group and 177 in the ICNB group) evaluated postoperative analgesic consumption. Through pooled analysis, no statistically significant difference (MD = 0.14, 95% CI: −1.04 to 1.32, *p* = 0.81) was found between the two groups, indicating that ESPB and ICNB are comparable in terms of postoperative analgesic requirements.

Moderate heterogeneity was noticed across the selected studies (*I* ^2^ = 57%, *p* = 0.04), reflecting notable variability in study outcomes. Fiorelli et al. (2020) reported a slight significant reduction (MD = −0.39, 95% CI: −0.74 to −0.04) in analgesic use with ESPB, while Gams et al. (2023) showed a notably significant MD (MD = −16.70, 95% CI: −29.68 to −3.72) favouring ESPB but with an extensive CI, indicating imprecision. None of the remaining studies reported any significant findings concerning analgesic consumption, as shown in Figure 3.

**FIGURE 3:**
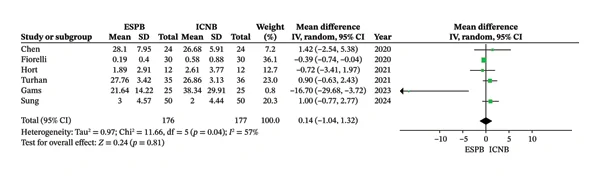
Forest plot of analgesic consumption.

For sensitivity analysis, the leave‐one‐out approach was used to determine the effect of the respective studies on the pooled calculation. Exclusion of *Fiorelli* et al. changed the pooled effect estimate from (MD = 0.14, 95% CI: −1.04 to 1.32, *p* = 0.81) to (MD = 0.35, 95% CI: −1.38 to 2.08, *p* = 0.69), while the overall result remained nonsignificant. Heterogeneity decreased slightly from *I* ^2^ = 57% to *I* ^2^ = 52%, suggesting that the results were not due to any individual study.

## 4. Discussion

This systematic review and meta‐analysis compares the analgesic efficacy of the ESPB and the ICNB in patients undergoing thoracoscopic surgery and reports these key findings. The primary outcome, i.e., dynamic postoperative pain scores at 48 h, is reported by five out of six included studies, which indicate that the ESPB is correlated with lower postoperative pain scores in contrast to ICNB, although the observed effect size was small. To further interpret the clinical relevance of this finding, it is important to consider the minimal clinically important difference for postoperative pain scores. Previous research has shown that a reduction of 1–2 points on the VAS or NRS is generally needed to be considered clinically meaningful [17, 18]. In this context, although the SMD observed (−0.34) is statistically significant, it likely represents a small effect that may not translate into a perceptible improvement in patient‐reported pain outcomes. Secondly, both ESPB and ICNB are comparable in terms of postoperative analgesic requirements.

The findings of the present study are supported by a previous meta‐analysis by Ma et al. [13]. However, the current study incorporates two additional RCTs, which increase the pooled sample size to 353 from 203. The incorporation of the most recent evidence provides an updated assessment of the comparative analgesic efficacy of both ESPB and ICNB for thoracoscopic surgery. Despite this, the overall conclusion remained similar, with ESPB demonstrating lower pain scores at 48 h but reporting no significant difference in postoperative analgesic consumption. The consistency across both studies reinforces the available evidence on the effectiveness of ESPB for pain relief during thoracoscopic procedures. However, despite the relatively small effect size and the absence of a significant opioid‐sparing effect, these findings should be interpreted for their clinical relevance rather than their statistical significance.

This modest decrease in pain score with ESPB may be due to its ability to offer broader, well‐sustained segmental analgesia from a single injection site, hence improving patient comfort, especially during movement in the early postoperative period. In contrast, ICNB typically requires multiple‐level injections and may provide only limited or shorter‐duration analgesia [4, 21, 22]. However, given the small magnitude of the effect observed and the absence of differences in postoperative analgesic consumption, these theoretical advantages appear not to translate to a clinically meaningful superiority of ESPB over ICNB.

The paradoxical findings with respect to the lack of association between low postoperative pain scores and lack of significant reduction in postoperative analgesic use warrant further discussion. There are multiple factors that contribute to analgesic consumption other than pain score, including hospital analgesic guidelines, predefined analgesic rescue levels, individual pain threshold level, and prescribing habits of anaesthesiologists. Therefore, reduced pain scores do not necessarily correlate with differences in the requirement of opioids and nonopioids. Moreover, there were different approaches to multimodal analgesia and postoperative pain management among the analysed studies, possibly leading to a reduction in the opioid‐sparing effect due to ESPB. This demonstrates that analgesic consumption needs to be interpreted in relation to patient‐reported pain outcomes, but not as a sole parameter of block effectiveness. Future RCTs using standardized postoperative analgesic protocols and uniform reporting of opioid consumption in morphine milligram equivalents may provide a more accurate assessment of the comparative opioid‐sparing potential of ESPB and ICNB.

There was moderate heterogeneity in analyses of postoperative analgesic consumption, which may be partly attributable to differences in analgesic protocols across the included trials. In the publication by Gams et al., we noticed that the patients in the ESPB group were given continuous infusion of local anaesthetic, in addition to an initial bolus. However, in the ICNB group, a single‐shot technique was used [12]. Such a discrepancy between how blocks are delivered and the subsequent design of postoperative analgesia would most likely affect the outcome in terms of analgesic consumption and reduce comparability between studies, thereby contributing to heterogeneity in the pooled analysis. Moreover, heterogeneity may also arise from differences in rescue analgesia protocols across studies, variability in opioid administration thresholds, and inconsistencies in reporting units of analgesic use. In addition, variations in perioperative analgesic regimens and the use of multimodal pain management approaches during the postoperative period may affect postoperative analgesic requirements and further preclude direct comparisons between studies.

In a clinical setting, these results imply that both ESPB and ICNB can still be considered viable regional analgesic approaches in thoracoscopic surgeries. While ESPB showed a statistically significant decrease in postoperative pain score values, no decrease in analgesic use suggests that the difference in the overall clinical benefit might not be substantial. In this regard, the choice of regional analgesic approach should take into account several criteria, including anaesthesiologist experience, availability of ultrasound, institutional standards, patient‐specific characteristics and duration of postoperative analgesia. Further studies should include outcomes such as patient satisfaction, mobilization timelines and functional recovery to better compare the clinical value of both techniques beyond conventional pain scores and opioid consumption.

Out of all the included studies, *Fiorelli* et al. reported a notably stronger analgesic effect for ESPB than any other included studies [8], indicating that this single study might have a disproportionate impact on the pooled estimate. For this purpose, leave‐one‐out sensitivity analyses were conducted. Although exclusion of this study diminished the magnitude of the pooled effect, the overall orientation of the observed conclusions did not change; therefore, these results must be interpreted cautiously and are susceptible to study‐level heterogeneity.

When interpreting these findings, it is crucial to consider the clinical heterogeneity among the included studies, particularly with regard to modifications in block techniques (i.e., differences in injection levels and ultrasound guidance, as well as local anaesthesia volume and concentration). Furthermore, whether the block is injected before or after the surgical procedure may influence both pain perception and subsequent analgesic requirements. These factors introduce variability across studies and may partially account for differences observed in treatment effects.

There are several limitations of this study that should be acknowledged. Firstly, this study included only a small number of RCTs with relatively small sample sizes, which may reduce the generalizability of the current findings to a broader patient population and increase the possibility of overestimating treatment effects. Additional limitations include variable randomization and limited or no blinding across included trials, which may lead to performance and detection bias. Variability in the included thoracoscopic procedures adds to clinical heterogeneity that could impact postoperative pain outcomes. Additionally, postoperative opioid consumption could not be uniformly converted to morphine milligram equivalents, and therefore, direct comparability of these studies is limited. The varied analgesic protocols across the included RCTs, including the use of different local anaesthetics such as bupivacaine, ropivacaine, and levobupivacaine, at different concentrations and dosing frequencies, further contribute to heterogeneity. Differences in the timing of block administration, such as preoperative versus postoperative, may also have altered the pain outcomes. Assessment of pain at a single time point, such as 48 h, limits the evaluation of the long‐term analgesic efficacy and adverse effects. Finally, one of the included studies (Horth et al.) was designed as a feasibility trial, which may provide less robust evidence compared with fully powered RCTs [7].

Another important consideration is the variability in outcome reporting across the included RCTs. Although postoperative pain scores and analgesic consumption were the most commonly assessed, there was inconsistency or no information on other important outcomes, such as patient satisfaction, quality of recovery, pulmonary function, duration of hospitalization, and return to normal activities. These outcomes are especially significant in thoracic surgery since good postoperative analgesia can not only help in pain reduction but also improve respiratory function, earlier mobilization, and recovery. Hence, future studies need to apply standardized methods for the evaluation of these outcomes.

Therefore, establishing the definitive postoperative analgesic efficacy of the ESPB in diverse surgical procedures calls for further high‐quality standardized RCTs. While narrative reviews have underscored the expanding clinical use of ESPB beyond thoracic surgery as part of multimodal analgesic strategies [22], evidence from emerging procedure‐specific studies has demonstrated its effectiveness in reducing postoperative pain and improving patient satisfaction in nonthoracic surgeries, such as percutaneous nephrolithotomy [23]. Nevertheless, the existing literature is heterogeneous, and future trials with larger sample sizes, adequate blinding, consistent outcome reporting, and longer follow‐up are required to verify the comparative efficacy, potential opioid‐sparing effects, and impact on chronic postoperative pain.

## 5. Conclusion

The results of this updated systematic review and meta‐analysis indicate that the ESPB is associated with a lower dynamic pain score at 48 h following a thoracoscopic operation in contrast to the ICNB. However, the estimated difference represents a small effect size, and both techniques showed comparable postoperative analgesic consumption. Given the small effect size, moderate heterogeneity observed in analgesic consumption outcomes, limited sample sizes, and variability in surgical procedures and analgesic protocols across the included studies, these findings should be interpreted carefully. Further large‐scale, well‐designed RCTs are required to better elucidate the clinical relevance and major contribution of the ESPB for postoperative pain reduction following thoracic surgery.

## Author Contributions

Muhammad Shaheer Bin Faheem: conceptualization, validation, visualization, software, formal analysis, project administration, supervision, writing–original draft, and writing–review and editing. Syeda Umbreen Munir: data curation, formal analysis, visualization, and writing–review and editing. Kanz Ul Eman Maryam: investigation, writing–original draft, and resources. Sabahat Ul Ain Munir Abbasi: methodology, validation, and resources. Sumaya Samadi: investigation, methodology, and resources.

## Funding

This research was not granted any specific funds from funding agencies in the public, commercial, or not‐for‐profit sectors.

## Ethics Statement

The present study does not involve direct data collection from human subjects or animals. All data used in this study were obtained from previously published and publicly available sources, adhering to the ethical guidelines of the respective studies. No identifiable patient information is presented in this study.

As this study is a systematic review and meta‐analysis of previously published randomized controlled trails and did not involve any direct participation of human subjects, institutional review board/institutional ethics committee approval was not required for the present study. Ethical approval had been obtained in all included randomized controlled trials according to their respective institutional policies.

## Consent

The authors have nothing to report.

## Conflicts of Interest

The authors declare no conflicts of interest.

## Supporting Information

Additional supporting information can be found online in the Supporting Information section.

## Supporting information


**Supporting Information** Supporting Table S1: Pubmed Search strategy. Supporting Figure S1: Prisma 2020 Checklist. Supporting Table S2: Quality Assessment of included studies.

## Data Availability

The datasets generated and/or analysed during the current study are available from the corresponding author on reasonable request.
