# Supplementary material for: An Updated Meta‐Analysis of Randomized Controlled Trials Comparing the Analgesic Efficacy of Intercostal Nerve Block Versus Erector Spinae Plane Block in Thoracic Surgery
Source: Anesthesiol Res Pract. 2026 Nov 3;2026:7833175. doi: 10.1155/anrp/7833175 (PMC13729267; doi:10.1155/anrp/7833175)
Supplement: Supplementary file 1 [file ANRP-2026-7833175-s001.zip › Supplementary file 1.docx]

**Supplementary Table S1:** Pubmed Search strategy

| Database | Search Strategy |
| --- | --- |
| Pubmed | ("thoracic surgery" OR "video-assisted thoracic surgery" OR VATS OR "minimally invasive thoracic surgery" OR "thoracoscopic surgery" OR "thoracoscopy" OR "thoracic procedure" OR "lung surgery" OR "chest surgery")AND("erector spinae plane block" OR ESPB OR "ultrasound-guided erector spinae plane block" OR "ESP block" OR "erector spinae block" OR "paravertebral block" OR "thoracic nerve block" OR "regional anesthesia" OR "regional analgesic technique")AND("intercostal nerve block" OR "thoracic paravertebral block" OR "intercostal block" OR "nerve block" OR "regional block" OR "anesthetic block") |

**Supplementary Table S2:** Quality Assessment of included studies
